# Endometriosis and Pregnancy: A Single Institution Experience

**DOI:** 10.3390/ijerph17020401

**Published:** 2020-01-08

**Authors:** Maria Grazia Porpora, Federica Tomao, Adele Ticino, Ilaria Piacenti, Sara Scaramuzzino, Stefania Simonetti, Ludovica Imperiale, Chiara Sangiuliano, Luisa Masciullo, Lucia Manganaro, Pierluigi Benedetti Panici

**Affiliations:** 1 Department of Maternal and Child Health and Urological Sciences, Sapienza, University of Rome, Policlinico Umberto I, Viale del Policlinico 155, 00161 Rome, Italy; mariagrazia.porpora@uniroma1.it (M.G.P.); Federica.tomao@uniroma1.it (F.T.); adele.ticino@yahoo.com (A.T.); chiara.sangiuliano@uniroma1.it (C.S.); luisa.masciullo@uniroma1.it (L.M.); pierluigi.benedettipanici@uniroma1.it (P.B.P.); 2 Department of Gynecology and Obstetrics Hôpital Erasme, Université Libre de Bruxelles, Route de Lennik, 808-B-1070 Brussels, Belgium; Ludovica.imperiale@erasme.ulb.ac.be; 3 Department of Radiological, Oncological, and Pathological Sciences, Sapienza, University of Rome, Policlinico Umberto I, Viale del Policlinico 155, 00161 Rome, Italy; lucia.manganaro@uniroma1.it

**Keywords:** endometriosis, adenomyosis, obstetric complications, caesarean section

## Abstract

Endometriosis may compromise the physiological course of pregnancy. The aim of this prospective observational study was to evaluate whether endometriosis causes a higher prevalence of obstetric and neonatal complications as well as a higher risk of caesarean section and to detect a possible correlation between the presence, type, and location of endometriosis and obstetric complications, previous surgery, and pregnancy outcome, as well as the influence of pregnancy on the course of the disease. We compared two cohorts of women with spontaneous pregnancy, with and without endometriosis. Obstetric and neonatal outcomes, mode of delivery, presence, type, and location of endometriotic lesions and the effect of pregnancy on the disease were analyzed. A total of 425 pregnancies were evaluated: 145 cases and 280 controls. Patients with endometriosis showed a higher incidence of miscarriage, threatened miscarriage, threatened preterm labor, preterm delivery, placental abruption, and a higher incidence of caesarean section. A significant correlation with pregnancy-induced hypertension and preeclampsia was found in the presence of adenomyosis. No difference in fetal outcome was found. One case of hemoperitoneum during pregnancy was observed. Pregnancy in women with endometriosis carries a higher risk of obstetric complications, such as miscarriage, threatened miscarriage, preterm labor, preterm birth, and a higher caesarean section rate. Endometriosis does not seem to influence fetal well-being.

## 1. Introduction

Endometriosis is a chronic inflammatory disease characterized by the presence of endometrial-like tissue outside the uterus in pelvic and extrapelvic locations [1]. The prevalence rate of endometriosis in perimenopausal women is about 10% [2,3]. The disease is associated with pelvic pain in 60–80% of cases and infertility in 30–40% of cases [4]. Although there is strong evidence of connection between endometriosis and infertility, a causal relation cannot always be demonstrated [5]. In advanced stages, correlation could be due to anatomical distortion, whereas immunological disorders and inflammation could play an important role in the early stages [6]. It has been reported that infertile women with ovarian endometrioma might also have uterine artery blood flow alterations which improve after surgery, thereby increasing pregnancy rate [7]. Spontaneous pregnancy occurs in about 40% of women who have undergone surgery [8]. Laparoscopic treatment of endometriosis enhances fertility and the highest pregnancy rate is generally observed within the first six months after surgery [9]. Several authors have recently focused their attention on the possible role of endometriosis in the occurrence of obstetric complications. Hormonal and immunological factors characterizing the disease seem to be involved in these complications [10]. In addition, in women affected by endometriosis and adenomyosis, changes in the uterine environment and junctional zone alterations seem to influence decidualization, placentation, uterine contractility, cervical maturation, and membrane disorders with consequent development of obstetric complications [10,11,12]. Several studies have assumed that endometriosis may increase the risk of preterm birth and premature rupture of membranes [13], as well as placental abruption and placenta previa [14]. The relationship between endometriosis and pregnancy-induced hypertension/preeclampsia is still being debated [15,16]. Conflicting data on the risk of miscarriage have been reported [17,18]; moreover, some authors found a higher incidence of miscarriage in women with ovarian endometriosis compared to other locations of the disease [19]. An increased caesarean section rate in women affected by endometriosis compared to healthy controls has been reported [20]. Furthermore, some authors hypothesize that pregnancy could induce maternal complications such as bowel perforation and intestinal bleeding in patients with intestinal location, or uroperitoneum in patients with urinary tract involvement. Endometriosis is also considered a major risk factor for spontaneous hemoperitoneum in pregnancy due to the bleeding of decidualized endometriotic foci [10]. The aim of our study was to examine the effects of endometriosis on pregnancy outcome, comparing women with and without endometriosis, and the effect of pregnancy on the course of the disease. The primary endpoints were pregnancy, neonatal outcome, and mode of delivery. Secondary endpoints were the evaluation of a possible association between adverse pregnancy outcomes and the presence, type, and location of endometriotic lesions diagnosed during pregnancy and those removed at previous surgery. Another endpoint was the evaluation of maternal complications occurring due to the impact of pregnancy on endometriotic lesions.

## 2. Materials and Methods

This prospective cohort study was performed at our University hospital from June 2013 to March 2019. The study was approved by the Ethics Committee (protocol No. 982/17, CE code 4782). We enrolled 425 Caucasian pregnant women referred to the Department of Maternal and Child Health and Urological Sciences of the Umberto I University Hospital that met the inclusion criteria. The case group included 145 women affected by endometriosis and 280 controls not affected by the disease. Inclusion criteria were age 18 to 45 years, natural conception, informed consent to the study, Caucasian race, surgical/histological or clinical/instrumental diagnosis of endometriosis in the case group, and the absence of clinical and imaging signs or surgical history of endometriosis in the control group. Exclusion criteria were assisted reproductive technology (ART) to conceive, multiparity, intention of pregnancy termination, increased obstetric risks (i.e., antiphospholipid syndrome, chronic hypertension, pre-existing diabetes, autoimmune diseases, congenital and uncorrected uterine abnormalities), smoking, alcohol and/or drug addiction during and before pregnancy. The choice to include only first pregnancies was made to reduce confounding factors, as a previous caesarean section could influence the type of delivery. In addition, we had only a few cases of subsequent pregnancies, and a comparison would not have allowed a statistical analysis. After a careful assessment of eligibility criteria, informed consent was obtained from all patients. Both groups underwent a monthly clinical and ultrasound examination, as well as blood tests until delivery. For each patient, age, body mass index (BMI), and obstetrical data were recorded. All clinical data were collected in an appropriate database. Maternal outcomes of interest included: miscarriage, threatened miscarriage, ectopic pregnancy, preterm birth, threatened preterm labor, premature rupture of membranes, pregnancy-induced hypertension, preeclampsia, intrahepatic cholestasis, placenta previa, placenta accreta, placental abruption, intrauterine growth restriction, gestational diabetes, and oligohydramnios. Gestational age, expressed in weeks and days of amenorrhea, was calculated from the first day of the last menstrual period. The preterm subgroup was classified as very low preterm <28 weeks, low preterm ≥28 to <32 weeks, moderate preterm ≥32 to <34 weeks, and late preterm ≥34 to <37 weeks. Mode of delivery (vaginal delivery, caesarean section) was recorded, and the occurrence of post-partum hemorrhage (maternal blood loss estimated to be >500 mL within 24 h of vaginal delivery or >1000 mL after caesarean section) was evaluated. Data collected from newborns were birth weight and Apgar score. According to their weight, newborns were divided into three groups: <1500 g, between 1500 and 2500 g, and > 2500 g; Apgar score >7 or <7 at 5 min was assessed. Obstetric outcome analysis on spontaneous single and multiple pregnancies and the number of live births and stillbirths was also assessed. Among patients in the case group, presence, type, and site of endometriosis were recorded (ovarian endometrioma, deep infiltrating endometriosis (DIE), adenomyosis, extrapelvic endometriosis), previous surgery, and the stage of disease according to Revised American Society for Reproductive Medicine (r-ASRM). The above parameters were correlated with the possible occurrence of obstetric complications. Finally, we evaluated the impact of pregnancy on the course of the disease and the possible onset of complications most frequently described in the literature, such as uro/hemoperitoneum and intestinal perforation.

Analysis of data was performed using the IBM SPSS Statistics Package (version 24, provided by “Sapienza” University, Rome, Italy). In order to study the characteristics of the two different populations and compare the mean values of independent variables, the Student’s T test was applied. The Chi-square test and the Pearson correlation coefficient were used to compare the outcomes in the two groups, and Fisher’s exact test in the case of very low absolute frequencies. A *p*-value of <0.05 was considered to indicate statistical significance.

## 3. Results

The study population included a total of 425 pregnant women who conceived spontaneously: 145 affected by endometriosis and 280 control patients not affected by endometriosis. The two cohorts were homogeneous in terms of BMI, parity, and twin pregnancy. There was a significant difference in median age, with the cases older than the controls (median 31 years versus 29 years; *p* < 0.0001); however, multivariate analysis did not show an influence of age on the results (Table 1).

In the case group, 73% of patients (106) had surgically/histologically confirmed endometriosis: 16 patients, stage I–II; and 90, stage III–IV (15% and 85%, respectively); characteristics of the lesions are reported in Table 2. Thirty-nine women (27%) did not undergo surgery and diagnosis was based only on clinical and imaging evaluation (ultrasound/magnetic resonance imaging) (Table 2).

During pregnancy, an ovarian endometrioma was present in 86% (125) of cases. DIE was present in 12% of patients (18); of these, nine patients had bowel endometriosis, six bladder localization, and three vaginal nodules. Extrapelvic endometriosis was present in 16% of patients (24); of these, five patients had abdominal wall endometriosis, one patient had inguinal localization, and one woman had diaphragmatic endometriosis. Adenomyosis was present in 11% of patients (16) (Table 2).

Women with endometriosis showed a significantly higher incidence rate of miscarriage (21%, 30) (*p* < 0.004), threatened miscarriage (5%, 7) (*p* = 0.036), threatened preterm labor (9.6%, 14) (*p* = 0.014), preterm birth (20%, 29) (*p* < 0.001). No significant difference in premature rupture of membranes, fetal growth restriction, pregnancy-induced hypertension, preeclampsia, gestational diabetes, intrahepatic cholestasis, oligohydramnios, placenta previa, placenta accreta, placental abruption, stillbirth, post-partum hemorrhage, and post-term pregnancy rates was observed. A higher incidence rate of ectopic pregnancy was found in the endometriosis group, however the difference between cases and controls was not statistically significant (*p* = 0.09) (Table 3).

An evaluation of the relationship between type of delivery and neonatal outcome was performed in a total of 361 patients, considering only ongoing evolutive pregnancies (111 cases versus 250 controls), excluding cases of miscarriage or extrauterine pregnancy. This analysis showed a higher incidence rate of caesarean section in the case group compared to the control group: caesarean section 35% versus 31% (51 versus 87) and vaginal birth 41% versus 58% (60 versus 163) *p* = 0.042 (Table 3). Stillbirth occurred in 1% (2) of cases and 0.4% (1) of controls (*p* = Ns). In the case group, preterm delivery occurred in 29 (20%) patients (including a twin pregnancy), mostly late-preterm newborns, while preterm delivery occurred in 20 (8%) of the control group. This difference was statistically significant (*p* < 0.001). There were no significant fetal complications among the preterm babies. No significant difference was found regarding birth weight and Apgar score at 5 min in both term and preterm newborns (Table 4).

Our study showed that the type and site of endometriosis, pre-existing or occurring during pregnancy (ovarian endometrioma, DIE, or extrapelvic endometriosis), did not influence pregnancy outcome, whereas a significant correlation was observed between the presence of adenomyosis and pregnancy-induced hypertension (7 pts 24% versus 1 pt 3%; *p* < 0.0001) and/or preeclampsia (3 pts, 10% versus 1 pt 3%; *p* < 0.0001).

As to possible maternal complications in women affected by endometriosis, only one case of spontaneous hemoperitoneum due to endometrioma rupture was observed. Analysis of the results in patients who underwent laparoscopy before pregnancy and in those who had no previous surgery showed that the removal of endometriotic tissue did not significantly influence obstetrical outcomes.

## 4. Discussion

Due to sex hormonal changes, pregnancy and lactation have been considered for over a century to have beneficial effects on patients with endometriosis. Recent studies, however, have shown that pregnancy does not seem to have a systematically favorable effect on women with endometriosis [21]. On the contrary, they suggest that the disease could interfere with the physiological course of pregnancy and be involved in a wide spectrum of early obstetric complications, such as miscarriage and ectopic pregnancy, as well as late pregnancy complications such as severe preeclampsia, hemorrhage during pregnancy, placental abruption, placenta previa, premature rupture of membranes, preterm birth, infants small for gestational age, and caesarean delivery [20,22,23]. Our study showed that endometriosis may lead to an increased incidence of miscarriage, threatened miscarriage, threatened preterm labor, preterm birth, placental abruption, and caesarean section. However, we found no correlation between the type and site of the disease and obstetrical complications. In patients with endometriosis, the risk of unfavorable obstetrical outcome seems to be increased in the first pregnancy compared to subsequent pregnancies. In fact, a study conducted by Conti et al. showed a higher risk of pProm (preterm prelabour rupture of membranes) and PTB (preterm birth), in line with our results, but also of SGA (small for gestational age) babies and Neonatal Intensive Care Unit (NICU) admission at birth [24].

Multiple mechanisms have been hypothesized to explain these pregnancy complications, such as impaired endometrial receptivity, decidualization, and remodeling of the uterine spiral vessels, which have been reported in patients with endometriosis. Inadequate endometrial receptivity caused by progesterone resistance and altered uterine contractility may provoke miscarriage [25,26]. Vercellini et al. found a higher miscarriage rate in nulliparous women with spontaneous pregnancies in the presence of ovarian endometrioma with or without peritoneal endometriosis (OR = 1.70). This association could be related to a genotoxic effect on the oocyte induced by the iron content in the endometrioma fluid [19]. Schwartz et al. reported a higher miscarriage rate in patients with mild endometriosis, as superficial lesions provoke severe inflammation, possibly leading to defective folliculogenesis, fertilization, and/or implantation [27]. A retrospective cohort study thus showed a higher miscarriage rate in all types of endometriosis (superficial, endometrioma, DIE) compared to the healthy controls [28]. In most studies the results are not changed by the mode of conception, and this constitutes a major confounding factor. In the present study only patients with spontaneous pregnancy were enrolled.

Our study showed a strong correlation between endometriosis and preterm birth, mainly late preterm, whereas no correlation was found between endometriosis and premature rupture of membranes. Increased expression of inflammatory pathways, prostaglandins, and activation of metalloproteinase causing cervical ripening and collagen degradation, uterine contractions, and inflammation of the membranes have been reported [11,29]. These mechanisms could explain the occurrence of preterm birth/premature rupture of membranes in patients with endometriosis. Recent studies found that preterm birth rate increases independently of the pregnancy onset (spontaneous or after ART) [14,30,31]. A significant correlation between preterm birth/premature rupture of membranes and adenomyosis [13,32] and DIE has recently been reported [33]. However, hormonal changes related to pregnancy may also have a protective role, as the rise of progesterone levels may reduce the typical inflammation caused by endometriosis, thereby reducing obstetrical complications in subsequent pregnancies [24].

We found an increased incidence of caesarean sections in the case group compared to controls, mainly performed in elective regimen. Several meta-analyses and reviews found a correlation between endometriosis and caesarean section [29,34]. In a study performed by Maggiore et al., the main indication for caesarean section in women with endometriosis was fetal distress, followed by breech presentation and dystocia [26]. Abnormal placentation in women with endometriosis may increase the risk of placental complications, antepartum hemorrhage, and preterm birth, thereby increasing the caesarean section rate [14,35]. An Italian retrospective study found that 36.6% of patients with a history of surgery for endometriosis underwent caesarean section, mostly women with ovarian and rectovaginal endometriosis [19]. Other authors reported an increased risk of caesarean section in women with DIE of the anterior and posterior compartment, regardless of previous surgery [36,37]. Despite the reported correlation between adenomyosis and caesarean section due to fetal malpresentation, probably caused by narrowing and reduced uterine extensibility [38], no correlation between disease site and caesarean section was observed in our study. In a large Swedish cohort study, elective caesarean section was more common than emergency operation in primipara with endometriosis [14]. In the literature, elective caesarean section is more frequent in patients with endometriosis, also excluding pregnancies after ART and those complicated by preeclampsia, preterm birth, or intrauterine growth restriction [39]. The high rate of caesarean sections in women with endometriosis may be due to, e.g., psychological factors, as women may choose to avoid pain during a vaginal birth and ask the physician to perform a caesarean section [39,40].

The risk of pregnancy-induced hypertension and preeclampsia in women with endometriosis is a controversial issue; some studies have reported that the risk of pregnancy-induced hypertension/preeclampsia in women with endometriosis has decreased [15], others report that it is unchanged [16] or increased [14,41]. The mechanism involved seems to be a defective remodeling of the spiral arteries in the junctional zone and in the placental bed, probably due to immunological disorders, chronic inflammation, and oxidative stress [42]. The possible correlation with the site of the lesions is also debated: Vercellini et al. did not find any correlation with the sites of the disease [19], while Exacoustos et al. reported an increased incidence of pregnancy-induced hypertension in the group of women with deep rectovaginal endometriosis [36]. A recent Japanese study found a significant correlation between pregnancy-induced hypertension/preeclampsia and diffuse adenomyosis. The authors hypothesized that chronic inflammation induced by adenomyosis and the thickness of the junctional zone could lead to abnormal placentation, resulting in hypertensive disorders [43,44].

Our results did not show any significant correlation between endometriosis and pregnancy-induced hypertension/preeclampsia, while a correlation with the presence of adenomyosis was found. A larger sample is required to confirm these results.

A few studies have analyzed neonatal outcome in women with endometriosis and some of them reported a higher incidence of small for gestational age newborns or low birth weight babies in affected women compared to the healthy controls [30]. This association was not correlated with the type of reproduction (spontaneous or ART) [30,37]. The typical inflammatory status that characterizes endometriosis may cause abnormalities in decidualization, leading to a poor placentation that may reduce nutrition and oxygenation of the fetus, with the risk of fetal growth restriction and SGA babies. Furthermore, as previously discussed, cytokines and other inflammatory molecules may be responsible for preterm delivery, with major risks of negative fetal outcomes, linked to prematurity [30]. Korelahti et al. did not find differences in birth weight, Apgar score, umbilical cord, pH value, and admission to an intensive care neonatal unit [45]. Only one study observed a longer period of hospitalization in the neonatal intensive care unit because of a higher incidence of prematurity [24]. Most studies compared pregnancies in women with or without endometriosis, not considering the type of conception (spontaneous or ART). In particular, the studies included in the systematic review conducted by Bruun et al. did not distinguish elective from spontaneous preterm deliveries. All these confounding factors may have influenced the results and the correlation between endometriosis and poor fetal outcome [32]. In the present study, no significant difference was found between cases and controls regarding birth weight, Apgar score, and newborn health. These findings could probably be explained by the inclusion of only spontaneous pregnancies in our study. There was a statistically significant difference in the rates of preterm delivery between cases and controls. However, no significant fetal complications occurred in these newborns, probably because they were late preterm. Overall, no difference was found in fetal outcomes between cases and controls at any gestational age. Our results thus suggest that endometriosis seems not to have a negative effect on fetal well-being. Nonetheless, more studies are required to confirm these findings. Complications of preexisting endometriotic foci during pregnancy are rare [46]; in our study, we observed one case of hemoperitoneum due to probable rupture of an ovarian endometrioma in the third trimester of pregnancy. This complication may have been related to the process of involution of the decidualized ectopic endometrium, as already discussed in the literature by other authors [26,47,48].

## 5. Conclusions

Endometriosis may interfere with the physiological course of pregnancy, and physicians should therefore treat these patients with particular care. To the best of our knowledge, this is the largest prospective single-center study of patients with endometriosis and adenomyosis, including only first spontaneous pregnancies. However, this study has some limitations. First, the sample of patients with documented adenomyosis was too small for a correct interpretation of the results; secondly, the mechanisms involved were not studied. Further prospective studies are required to confirm our data, also evaluating the characteristics of placenta and chorion membranes in these patients. It is important that gynecologists inform women with endometriosis about possible obstetric complications. A careful maternal–fetal surveillance is needed in these patients.

## Figures and Tables

**Table 1:** Clinical characteristics of women with endometriosis and controls.

	Cases 145	Controls 280	*p*-Value
Maternal age (y)	31 × (18–45)	29 × (18–42)	<0.0001
BMI	22 × (17–35)	23 × (16–39)	Ns

Ns = not significant.

**Table 2:** Characteristics of endometriosis (145 patients).

**Type and Site of Endometriosis**	**Patients**	**%**
Endometriomas	125	86%
Peritoneal endometriosis *	66	45%
Extrapelvic endometriosis	24	16%
Deep infiltrating endometriosis	18	12%
Adenomyosis	16	11%
**Type of Diagnosis**	**Patients**	**%**
Clinical/instrumental	39	27
Surgery before pregnancy	106	73%
Stage I–II	16	15%
Stage III–IV	90	85%

* Peritoneal endometriosis: appraisable only in patients who previously underwent surgery.

**Table 3:** Pregnancy outcomes in women with endometriosis and controls.

Pregnancy Outcomes	Cases 145	Controls 280	*p*-Value	r Pearson Coefficient
	**Patients (%)**	**Patients (%)**		
Miscarriage	30 (21%)	28 (10%)	0.004	0.15
Threat of miscarriage	7 (5%)	4 (1%)	0.051	0.10
Ectopic pregnancy	4 (3%)	2 (1%)	Ns	
**Gestational Age (weeks)**	**Patients (%)** 111 (77%)	**Patients (%)** 250 (89%)		
Full term (37–42 w)	79 (55%)	226 (81%)	<0.001	−0.28
Post-term (>42 w)	2 (1%)	3 (1%)	Ns	
Preterm (<37 w)	29 (20%)	21 (8%)	<0.001	0.18
Very low preterm (<28 w)	2 (2%)	0	Ns	
Low preterm (28–32 w)	0	3 (1%)	Ns	
Moderate preterm (32–34 w)	3 (3%)	4 (2%)	Ns	
Late preterm (34–37 w)	24 (twin) * (22%)	14 (6%)	Ns	
**Threatened Preterm Labor**	14 (9.6%)	10 (4%)	0.014	0.12
Twin pregnancy	1 (1%)	2 (1%)	Ns	
**Childbirth Method**	**Patients 111**	**Patients 250**		
	**Patients (%)**	**Patients (%)**		
Vaginal delivery	60 (41%)	163 (58%)	0.042	
Caesarean section	51 (35%)	87 (31%)		
**Obstetric Complications**	**Patients 145**	**Patients 280**		
Premature rupture of membranes	8 (5.5%)	9 (3.2%)	Ns	
Fetal growth restriction	1 (1%)	5 (2%)	Ns	
Pregnancy induced hypertension	7 (5%)	16 (6%)	Ns	
Preeclampsia	3 (2%)	2 (1%)	Ns	
Gestational diabetes	3 (2%)	11 (4%)	Ns	
Intrahepatic cholestasis	1 (1%)	5 (2%)	Ns	
Oligohydramnios	1 (1%)	8 (3%)	Ns	
Placenta previa	4 (3%)	3 (1%)	Ns	
Placenta accreta	0	1 (0.4%)	Ns	
Placental abruption	2 (1%)	0	Ns	
Post-partum hemorrhage	4 (3%)	3 (1%)	Ns	
Stillbirth	2 (1%)	1 (0.4%)	Ns	

* Presence of one twin pregnancy in the case group, with late-preterm delivery. Ns = not significant.

**Table 4:** Comparison of neonatal outcomes in women with endometriosis and controls.

	Cases Patients 111	Controls Patients 250	*p*-Value
Birth Weight (Grams)	3094	3139	Ns
Mean range	(978–4330)	(1100–4600)	Ns
Birth Weight (Grams)	Patients (%)	Patients (%)	
>2500	97 (87%)	223 (89%)	Ns
1500–2500	9 (8%)	23 (9%)	Ns
<1500	5 (4%)	4 (2%)	Ns
Apgar Score	**Patients 111 (%)**	**Patients 250 (%)**	
5 min > 7	97 (87%)	231 (92%)	Ns
5 min < 7	14 (13%)	19 (8%)	

Ns = not significant.

## References

[1] Eskenazi B., Warner M.L. (1997). Epidemiology of endometriosis. Obstet. Gynecol. Clin. N. Am..

[2] Giudice L.C., Kao L.C. (2004). Endometriosis. Lancet.

[3] Horton J., Sterrenburg M., Lane S., Maheshwari A., Li T.C., Cheong Y. (2003). Reproductive, obstetric, and perinatal outcomes of women with adenomyosis and endometriosis: A systematic review and meta-analysis. Hum. Reprod. Update.

[4] Vinatier D., Orazi G., Cosson M., Dufour P. (2001). Review Theories of endometriosis. Obstet. Gynecol..

[5] Pfeifer S., Fritz M., Goldberg J., McClure D., Lobo R., Thomas M., Widra E., Schattman G., Licht M., Collins J. (2012). Practice Committee of the American Society for Reproductive Medicine. Endometriosis and infertility: A committee opinion. Fertil. Steril..

[6] Hjordt H.M.V., Dalsgaard T., Hartwell D., Skovlund C.W., Lidegaard Ø. (2014). Reproductive prognosis in endometriosis. A national cohort study. Acta Obstet. Gynecol. Scand..

[7] Porpora M.G., Tomao F., Manganaro L., Yazdanian D., Fuggetta E., Piccioni M.G., Benagiano G. (2014). Impaired uterine artery flow associated with the presence of ovarian endometrioma: Preliminary results of a prospective study. J. Ovarian Res..

[8] Roman H., Chanavaz-Lacheray I., Ballester M., Bendifallah S., Touleimat S., Tuech J.J., Farella M., Merlot B. (2018). High postoperative fertility rate following surgical management of colorectal endometriosis. Hum. Reprod..

[9] Porpora M.G., Pultrone D.C., Bellavia M., Franco C., Crobu M., Cosmi E.V. (2002). Reproductive outcome after laparoscopic treatment of endometriosis. Clin. Exp. Obstet. Gynecol..

[10] Brosens I., Brosens J.J., Fusi L., Al-Sabbagh M., Kuroda K., Benagiano G. (2012). Risks of adverse pregnancy outcome in endometriosis. Fertil. Steril..

[11] Petraglia F., Arcuri F., De Ziegler D., Chapron C. (2012). Inflammation: A link between endometriosis and preterm birth. Fertil. Steril..

[12] Brosens I., Derwig I., Brosens J., Fusi L., Benagiano G., Pijnenborg R. (2010). The enigmatic uterine junctional zone: The missing link between reproductive disorders and major obstetrical disorder?. Hum. Reprod..

[13] Juang C.M., Chou P., Yen M.S., Twu N.F., Horng H.C., Hsu W.L. (2007). Adenomyosis and risk of preterm delivery. BJOG..

[14] Stephansson O., Kieler H., Granath F., Falconer H. (2009). Endometriosis, assisted reproduction technology, and risk of adverse pregnancy outcome. Hum. Reprod..

[15] Brosens I.A., De Sutter P., Hamerlynck T., Imeraj L., Yao Z., Cloke B., Brosens J.J., Dhont M. (2007). Endometriosis is associated with a decreased risk of pre-eclampsia. Hum. Reprod..

[16] Hadfield R.M., Lain S.J., Raynes-Greenow C.H., Morris J.M., Roberts C.L. (2009). Is there an association between endometriosis and the risk of preeclampsia? A population-based study. Hum. Reprod..

[17] Brosens I., Pijnenborg R., Benagiano G. (2013). Defective myometrial spiral artery remodelling as a cause of major obstetrical syndromes in endometriosis and adenomyosis. Placenta.

[18] Matalliotakis I., Cakmak H., Dermitzaki D., Zervoudis S., Goumenou A., Fragouli Y. (2008). Increased rate of endometriosis and spontaneous abortion in an in vitro fertilization program: No correlation with epidemiological factors. Gynecol. Endocrinol..

[19] Vercellini P., Parazzini F., Pietropaolo G., Cipriani S., Frattaruolo M.P., Fedele L. (2012). Pregnancy outcome in women with peritoneal, ovarian and rectovaginal endometriosis: A retrospective cohort study. BJOG.

[20] Zullo F., Spagnolo E., Saccone G., Acunzo M., Xodo S., Ceccaroni M., Berghella V. (2017). Endometriosis and obstetrics complications: A systematic review and meta-analysis. Fertil. Steril..

[21] Leeners B., Damaso F., Ochsenbein-Kölble N., Farquhar C. (2018). The effect of pregnancy on endometriosis-facts or fiction?. Hum. Reprod. Update.

[22] Saraswat L., Ayansina D.T., Cooper K.G., Bhattacharya S., Miligkos D., Horne A.W., Bhattacharya S. (2017). Pregnancy outcomes in women with endometriosis: A national record linkage study. BJOG.

[23] Berlac J.F., Hartwell D., Skovlund C.W., Langhoff-Roos J., Lidegaard Ø. (2017). Endometriosis increases the risk of obstetrical and neonatal complications. Acta Obstet. Gynecol. Scand..

[24] Conti N., Cevenini G., Vannuccini S., Orlandini C., Valensise H., Gervasi M.T., Ghezzi F., Di Tommaso M., Severi F.M., Petraglia F. (2015). Women with endometriosis at first pregnancy have an increased risk of adverse obstetric outcome. J. Matern. Fetal Neonatal Med..

[25] Al-Sabbagh M., Lam E.W., Brosens J.J. (2012). Mechanisms of endometrial progesterone resistance. Mol. Cell. Endocrinol..

[26] Leone Roberti Maggiore U., Ferrero S., Mangili G., Bergamini A., Inversetti A., Giorgione V., Viganò P., Candiani M. (2016). A systematic review on endometriosis during pregnancy: Diagnosis, misdiagnosis, complications and outcomes. Hum. Reprod. Update.

[27] Kohl Schwartz A.S., Wölfler M.M., Mitter V., Rauchfuss M., Haeberlin F., Eberhard M., von Orelli S., Imthurn B., Imesch P., Fink D. (2017). Endometriosis, especially mild disease: A risk factor for miscarriages. Fertil. Steril..

[28] Santulli P., Marcellin L., Menard S., Thubert T., Khoshnood B., Gayet V., Goffinet F., Ancel P.Y., Chapron C. (2016). Increased rate of spontaneous miscarriages in endometriosis-affected women. Hum. Reprod..

[29] Vannuccini S., Clifton V.L., Fraser I.S., Taylor H.S., Critchley H., Giudice L.C., Petraglia F. (2016). Infertility and reproductive disorders: Impact of hormonal and inflammatory mechanisms on pregnancy outcome. Hum. Reprod. Update.

[30] Perez-Lopez F.R., Villagrasa-Boli P., Munoz-Olarte M., Morera-Grau Á., Cruz-Andrés P., Hernandez A.V. (2018). Association Between Endometriosis and Preterm Birth in Women with Spontaneous Conception or Using Assisted Reproductive Technology: A Systematic Review and Meta-Analysis of Cohort Studies. Reprod. Sci..

[31] Harada T., Taniguchi F., Onishi K., Kurozawa Y., Hayashi K., Harada T. (2016). Obstetrical Complications in Women with Endometriosis: A Cohort Study in Japan. PLoS ONE.

[32] Bruun M.R., Arendt L.H., Forman A., Ramlau-Hansen C.H. (2018). Endometriosis and adenomyosis are associated with increased risk of preterm delivery and a small-for-gestational-age child: A systematic review and meta-analysis. Acta Obstet. Gynecol. Scand..

[33] Mannini L., Sorbi F., Noci I., Ghizzoni V., Perelli F., Di Tommaso M., Mattei A., Fambrini M. (2017). New adverse obstetrics outcomes associated with endometriosis: A retrospective cohort study. Arch. Gynecol. Obstet..

[34] Leone Roberti Maggiore U., Inversetti A., Schimberni M., Viganò P., Giorgione V., Candiani M. (2017). Obstetrical complications of endometriosis, particularly deep endometriosis. Fertil. Steril..

[35] Lin H., Leng J.H., Liu J.T., Lang J.H. (2015). Obstetric outcomes in Chinese women with endometriosis: A retrospective cohort study. Chin. Med. J..

[36] Exacoustos C., Lauriola I., Lazzeri L., De Felice G., Zupi E. (2016). Complications during pregnancy and delivery in women with untreated rectovaginal deep infiltrating endometriosis. Fertil. Steril..

[37] Allerstorfer C., Oppelt P., Enzelsberger S.H., Shamiyeh A., Schimetta W., Shebl O., Bernhard Mayer R. (2016). Delivery after operation for deeply infiltrating endometriosis. Biomed. Res. Int..

[38] Mochimaru A., Aoki S., Oba M.S., Kurasawa K., Takahashi T., Hirahara F. (2015). Adverse pregnancy outcomes associated with adenomyosis with uterine enlargement. J. Obstet. Gynaecol. Res..

[39] Glavind M.T., Forman A., Arendt L.H., Nielsen K., Henriksen T.B. (2017). Endometriosis and pregnancy complications: A Danish cohort study. Fertil. Steril..

[40] Stern J.E., Luke B., Tobias M., Gopal D., Hornstein M.D., Diop H. (2015). Adverse pregnancy and birth outcomes associated with underlying diagnosis with and without assisted reproductive technology treatment. Fertil. Steril..

[41] Pan M.L., Chen L.R., Tsao H.M., Chen K.H. (2017). Risk of gestational hypertension-preeclampsia in women with preceding endometriosis: A nationwide population-based study. PLoS ONE.

[42] Benagiano G., Brosens I., Habiba M. (2014). Structural and molecular features of the endomyometrium in endometriosis and adenomyosis. Hum. Reprod. Update.

[43] Tamura H., Kishi H., Kitade M., Asai-Sato M., Tanaka A., Murakami T., Minegishi T., Sugino N. (2017). Complications and outcomes of pregnant women with adenomyosis in Japan. Reprod. Med. Biol..

[44] Razavi M., Maleki-Hajiagha A., Sepidarkish M., Rouholamin S., Almasi‐Hashiani A., Rezaeinejad M. (2019). Systematic review and meta-analysis of adverse pregnancy outcomes after uterine adenomyosis. Int. J. Gynaecol. Obstet..

[45] Kortelahti M., Anttila M.A., Hippeläinen M.I., Heinonen S.T. (2003). Obstetric outcome in women with endometriosis—A matched case-control study. Gynecol. Obstet. Investig..

[46] Vigano P., Corti L., Berlanda N. (2015). Beyond infertility: Obstetrical and postpartum complications associated with endometriosis and adenomyosis. Fertil. Steril..

[47] Glavind M.T., Møllgaard M.V., Iversen M.L., Arendt L.H., Forman A. (2018). Obstetrical outcome in women with endometriosis including spontaneous hemoperitoneum and bowel perforation: A systematic review. Best Pract Res. Clin. Obstet. Gynaecol..

[48] Lier M.C.I., Malik R.F., Ket J.C.F., Lambalk C.B., Brosens I.A., Mijatovic V. (2017). Spontaneous hemoperitoneum in pregnancy (SHiP) and endometriosis-a systematic review of the recent literature. Eur. J. Obstet. Gynecol. Reprod. Biol..

